# Undifferentiated Pleomorphic Sarcoma of the Descending Colon: An Infrequent Occurrence

**DOI:** 10.7759/cureus.61346

**Published:** 2024-05-30

**Authors:** Pir Abdul Ahad Aziz Qureshi, Shah Zeb, Thordur Tryggvason, Arnar Þórisson

**Affiliations:** 1 Department of Radiology, Landspítali - The National University Hospital of Iceland, Reykjavík, ISL; 2 Faculty of Medicine, Háskóli Íslands, Reykjavík, ISL; 3 Department of Pathology, Landspítali - The National University Hospital of Iceland, Reykjavík, ISL

**Keywords:** colon cancer, primary undifferentiated pleomorphic sarcoma, colon, pleomorphic sarcoma of colon, ups

## Abstract

Undifferentiated pleomorphic sarcoma (UPS), formerly known as malignant fibrous histiocytoma (MFH), constitutes a significant subset of soft-tissue sarcomas. Despite its rarity, UPS poses substantial clinical challenges due to its aggressive behavior and propensity for distant metastasis. Here, we report a rare case of high-grade UPS located in the colon, a site exceptionally uncommon for this malignancy, in a 49-year-old woman. The case also underscores the importance of considering UPS in the differential diagnosis of colonic neoplasms. Understanding the clinical and pathological features of UPS in unusual locations like the large intestine is crucial for timely diagnosis and appropriate management strategies.

## Introduction

Sarcomas are a diverse group of malignant tumors arising from mesenchymal tissues, constituting merely 1% of malignancies in adults [1]. Among them, undifferentiated pleomorphic sarcoma (UPS), also referred to as malignant fibrous histiocytoma (MFH) previously, represents 28% of soft-tissue sarcomas, typically manifesting in the extremities, followed by the retroperitoneum [2,3]. However, primary UPS originating in the bowel is exceedingly rare, with only a few documented cases reported in the literature to date [4]. The etiology of UPS still remains incompletely elucidated. Nonetheless, various predisposing factors, such as genetic aberrations, exposure to chemoradiotherapy, chronic irritation, and lymphedema, have been implicated in its development. Furthermore, UPS predominantly affects males aged 60 to 80 years, demonstrating a propensity for aggressive behavior characterized by extensive local invasion and distant metastasis. Poor prognosis often ensues due to delayed diagnosis and limited therapeutic options, with intra-abdominal UPS exhibiting even worse outcomes compared to its extremity counterparts [1]. However, UPS originating in the large intestine is exceptionally rare. Our case report presents an unusual case of high-grade UPS located in the colon.

## Case presentation

A 49-year-old woman with no known prior co-morbidities presented in the emergency department of our hospital in May 2021 with complaints of fever and abdominal pain. She consumes a little alcohol occasionally (a couple of drinks on weekends) and has no history of smoking. On general physical examination, the patient had a fever of 37.7°C and abdominal pain and tenderness. The pain was most severe in the epigastric region and left lower quadrant of the abdomen. No signs of peritoneal irritation were observed on the general physical examination. Blood investigations were ordered, and the results are outlined in Table 1.

**Table 1:** Blood investigation results of the patient at the time of admission. TLC: total leukocyte count; Hg: hemoglobin; CRP: C-reactive protein; CEA: Carcinoembryonic antigen

Investigations	Values on admission	Reference Range
TLC	13 x 10^9^/L	4-10.5 x 10^9^/L
Hg	108 g/L	118-152 g/L
Platelets	1141 x 10^9^/L	150-400 x 10^9^/L
CRP	49 mg/L	<10 mg/L
CA 125	62.0 U/mL	35 U/mL
CA 19-9	0.8 U/mL	35
CEA	0.9 µg/L	4.6 µg/L

**Figure 1:**
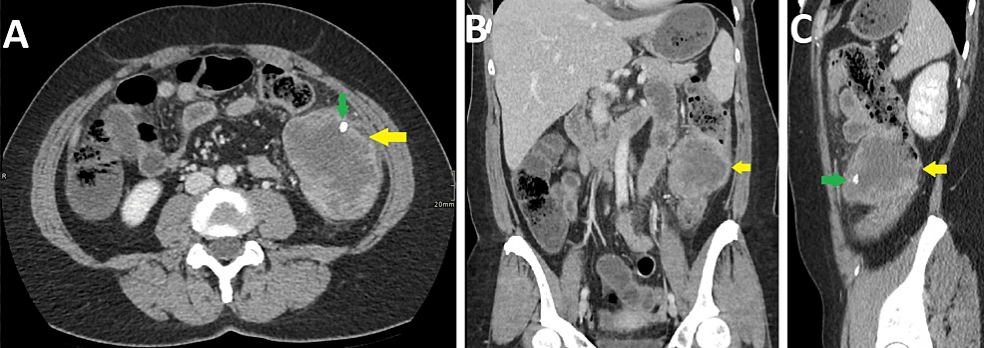
CT abdomen with contrast Axial view (A), Coronal view (B), and Sagittal view (C) show a large necrotic tumor involving the descending colon with an exophytic component (yellow arrow) and calcification within it (green arrow).

The patient was then advised to have a CT scan of the abdomen with contrast for further evaluation. Subsequently, the patient underwent a CT scan of the abdomen, which revealed a large necrotic lesion involving the descending colon measuring about 59 x 80 x 76 mm. The lesion showed a couple of small calcifications anterolaterally. The mass was associated with perifocal mild fat stranding and minimal free fluid. No enlarged abdominopelvic lymph nodes were identified. Based on the radiological findings, a necrotic tumor involving the descending colon was diagnosed (Figure 1).

**Figure 2:**
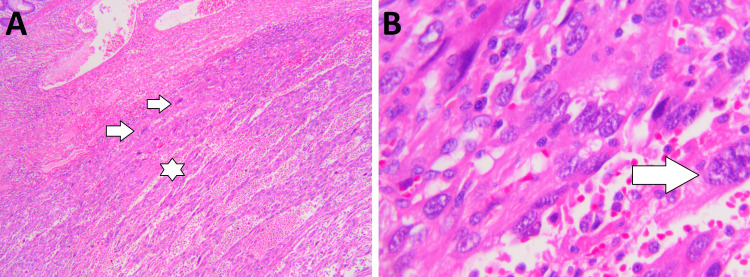
Histopathology of resected colon (A) 100x magnification, H&E staining. Sarcomatoid tumor growing into colon wall. Spindel cells (arrow) and epithelioid cells with necrosis (star) and bleeding. (B) 400x magnification, H&E staining. Monstrous giant cells in tumor (arrow) and pleomorphic tumor cells.

An additional CT thorax was also performed afterward to evaluate possible distant metastases, which turned out negative for distant metastasis. She then underwent a colonoscopic evaluation of the lesion, which showed a large irregular tumor with possible extraluminal growth. The biopsy was taken from the lesion, which was inconclusive. The patient was then discussed in the abdominal tumor board, and on the recommendations of the tumor board, the patient underwent an open left hemicolectomy. During the surgery, it was observed that the loops of the small intestine were also involved by the tumor; therefore, the surgery was extended to resect the involved part of the small bowel loops. The transverse colon was then reconnected to the sigmoid colon. The resected colon and small bowel loops were sent for histopathological evaluation, which showed a highly anaplastic tumor with a sarcomatoid appearance and prominent monstrous giant cells with highly atypical hyperchromatic nuclei and a very large number of mitosis, many of which are abnormal. Otherwise, the growth was made up of either epithelioid or spindle-shaped cells, and there is very pronounced necrosis in the tumor and hemorrhage. Furthermore, immunohistochemical stains were also performed on the tumor, which showed slightly positive staining for SMA and AE1/AE3 and minimal positive staining for CAM5.2. Vimentin was partially positive in the tumor but CD31 staining was insignificant. CK20, CK7, CDX2, desmin, caldesmon, Factor VIII, CD34, c-kit, myogenin, S100, and DOG1 were negative. Based on the histopathological evaluation, the diagnosis of undifferentiated pleomorphic sarcoma was made. Thirty-eight lymph nodes were also evaluated, and no tumor metastases were found (Figure 2). ​

Post-operatively, the patient experienced nausea, and her food intake was also reduced. Therefore, she was given parenteral nutrition. Otherwise, there were no post-operative complications, and the patient was discharged after 13 days of surgery and was put on regular weekly follow-ups. The patient was then again discussed in the tumor board for further management of the patient, which advised that the patient should undergo a follow-up CT scan at three months and a PET-CT scan at six months to evaluate recurrence. Subsequently, a CT scan was performed after three months of post-surgery, and the PET-CT scan after six months did not show disease recurrence. The patient did not receive any chemotherapy or radiotherapy since there was no residual or recurrent disease on the follow-up as per the decision of the tumor board. Now, on a three-year follow-up, the patient is doing well, without any active complaints or disease recurrence.

## Discussion

UPS, previously known as MFH, primarily affects the extremities (lower extremities - 49%; upper extremities - 19%) and less frequently the retroperitoneum (16%) [5]. It can involve various organs, including the head, neck, dura, brain, heart, lungs, pancreas, aorta, spleen, and liver, although visceral organ involvement is uncommon [4]. UPS in the colorectum predominantly affects male patients, with a male-to-female ratio of 2.5:1. Most cases presented as large tumors with varied symptoms, including abdominal pain, distention, anorexia, diarrhea, anemia, fever, and perineal pain. UPSs in the colon typically arise from deep fascia or muscularis and show extraluminal growth, often presenting with nonspecific clinical manifestations resembling colorectal cancer [6]. Colonoscopy may not detect abnormalities initially, but in cases of substantial tumor growth, it may reveal ulcerative lesions, hindering further examination. Diagnosis remains challenging, emphasizing the reliance on histopathology for definitive diagnosis. Immunohistochemical stains aid in the exclusion of other tumors, though no definitive immunophenotype for UPS subclassification exists [1].

The prognosis of UPS varies depending on the site of involvement; UPS of the extremities typically exhibits a poorer prognosis than abdominal involvement due to delayed diagnosis and challenges in biopsy accessibility [4]. Radical surgery remains the primary treatment for colorectal UPS, yet local recurrence or distant metastasis may occur despite extensive excision. The efficacy of adjuvant chemotherapy or radiotherapy is uncertain [1]. Analysis of previously reported cases and our case indicated a high rate of local recurrence or distant metastasis post-surgery, underscoring the poor prognosis of colorectal UPS. The survival rates at six months, one year, two years, and five years were 77.78%, 75.00%, 63.64%, and 12.50%, respectively [1]. Fortunately, our patient has no evidence of disease recurrence or distant metastases on a three-year follow-up. This highlights the importance of early diagnosis and management in such cases, which significantly impact the prognosis of the disease.

## Conclusions

In conclusion, UPS in the colorectum is a rare malignant tumor with an uncertain pathogenesis and poor prognosis. Radical surgery remains the cornerstone of treatment, with variable outcomes observed following adjuvant therapy. Radiologists are urged to maintain a high level of suspicion for this condition alongside more common malignant lesions, ensuring comprehensive consideration during diagnostic evaluations. Further research endeavors are warranted to deepen our understanding of UPS affecting the bowel, thereby enhancing clinical management strategies for this relatively rare yet clinically significant pathology.
